# Two-season effectiveness of a single nirsevimab dose against RSV hospitalisation in healthy term-born infants: a population-based case–control study, Spain, October 2023 to March 2025

**DOI:** 10.2807/1560-7917.ES.2026.31.9.2500593

**Published:** 2026-03-05

**Authors:** Olivier Núñez, Juan Juaneda, Montserrat Martinez-Marcos, Enriqueta Muñoz Platón, Eva Rivas Wagner, María-Isolina Santiago-Pérez, Virginia Álvarez Río, Matilde Zornoza Moreno, Ana Fernández Ibáñez, Gisselle Perez Suarez, Gorka Loroño Ortiz, Nerea Egüés, Belén Berradre Sáenz, María de los Ángeles Cuesta Franco, Susana Casado Cobo, María Domínguez Padilla, Daniel Castrillejo, Ana Míguez Santiyán, Luca Basile, María Ángeles Rafael de la Cruz López, Diana Sanabria Curbelo, Olaia Pérez-Martínez, M Jesús Rodríguez Recio, Lourdes Duro Gómez, María del Pilar Alonso Vigil, Manuel Mendez Diaz, Rosa Sancho, Jesús Castilla, Ana Carmen Ibáñez Pérez, Noa Batalla Rebollo, Lucía Sánchez Piorno, Ninoska López Berrios, Joaquín Lamas, Carmen Olmedo, Susana Monge, Roberto Pastor-Barriuso, the Nirsevimab Effectiveness Study Collaborators, The Nirsevimab Effectiveness Study Collaborators, Rocío Moreno Illueca, Katja Villatoro Bongiorno, Jacobo Mendioroz, Alba Moya, José Ramón Martínez Fernández, Carmen Román Ortiz, Rosa Álvarez-Gil, María-Teresa Otero-Barrós, Ana Treviño Nakoura, Kevin Javier Manzano Armas, Beatriz Bermejo Muñoz, María del Carmen Pacheco Martínez, Jaime Jesús Pérez Martín, Blanca Andreu Ivorra, Esteban Estupiñán Valido, Marta Huerta Huerta, Pello Latasa, Guillermo Ezpeleta, Manuel García Cenoz, Eva Martínez Ochoa, María Merino Díaz, Luis Javier Viloria Raymundo, Cristina Andreu Salete, Luisa Fernanda Hermoso Castro, Julián Manuel Domínguez Fernández, Sara Estefanía Montenegro Jaramillo

**Affiliations:** 1 National Centre of Epidemiology, Institute of Health Carlos III, Madrid, Spain; 2 Consortium for Biomedical Research in Epidemiology and Public Health (CIBERESP), Madrid, Spain; 3 Directorate-General for Public Health, Valencia, Valencian Community, Spain; 4 Agència de Salut Pública de Catalunya (ASPCAT), Barcelona, Catalonia, Spain; 5 Directorate-General of Public Health, Toledo, Castilla-La Mancha, Spain; 6 Directorate-General of Public Health, Canary Islands Health Service, Santa Cruz de Tenerife, Canary Islands, Spain; 7 Directorate-General of Public Health, Department of Health, Santiago de Compostela, Galicia, Spain; 8 Directorate-General of Public Health, Department of Health, Valladolid, Castilla y León, Spain; 9 Directorate-General of Public Health and Addictions, Murcia, Region of Murcia, Spain; 10 Directorate-General of Public and Mental Health, Oviedo, Asturias, Spain; 11 Directorate-General of Public Health, Zaragoza, Aragón, Spain; 12 Department of Health, Vitoria-Gasteiz, Basque Country, Spain; 13 Instituto de Salud Pública y Laboral de Navarra – IdiSNA, Pamplona, Navarra, Spain; 14 Directorate-General of Public Health, Logroño, La Rioja, Spain; 15 Directorate-General of Public Health, Mérida, Extremadura, Spain; 16 Directorate-General of Public Health, Santander, Cantabria, Spain; 17 University Hospital of Ceuta, Ceuta, Spain; 18 Directorate-General of Public Health, Melilla, Spain; 19 Public Health Department, Ceuta, Spain; 20 University Hospital of Melilla, Melilla, Spain; 21 Vaccines Division, Directorate-General of Public Health, Ministry of Health, Madrid, Spain; 22 Consortium for Biomedical Research on Infectious Diseases (CIBERINFEC), Madrid, Spain; 23 The members of the group are listed under collaborators; * These authors contributed equally to the work and share last authorship.

**Keywords:** Respiratory syncytial virus (RSV), Hospitalisation, Nirsevimab, Immunisation, Long-term effectiveness, Paediatric

## Abstract

**BACKGROUND:**

In autumn 2023, Spain recommended nirsevimab to all infants born after 1 April 2023, as catch-up or at-birth immunisation.

**AIM:**

We estimated effectiveness of a single nirsevimab dose against respiratory syncytial virus (RSV) hospitalisations throughout two seasons in healthy term-born infants.

**METHODS:**

Cases were children born 1 April 2023 through 31 March 2024 after 35 gestation weeks without major comorbidities and hospitalised for RSV infection between 2023 immunisation campaign onset and 31 March 2025. We selected four healthy population-density controls per case, matched by province and birth date. Using target trial emulation, causal per-protocol effectiveness was estimated for catch-up (within 30 days of 2023 campaign onset) and at-birth immunisation (within 14 days of life) through cloning, censoring and inverse-probability-weighted conditional logistic regression.

**RESULTS:**

We included 235/905 cases/controls for catch-up and 334/1,292 cases/controls for at-birth immunisation (first season), and 188/713 cases/controls for catch-up and 328/1,269 cases/controls for at-birth immunisation (second season). Two-season effectiveness was 64% (95% confidence interval (CI): 52–72) and 67% (95% CI: 59–74) for catch-up and at-birth immunisation, respectively, compared with 78% (95% CI: 70–84) and 84% (95% CI: 79–88) during first season and −8% (95% CI: −88 to 38) and 20% (95% CI: −21 to 46) during second season.

**CONCLUSION:**

Nirsevimab was an effective long-term population-level intervention, decreasing RSV hospitalisations by two-thirds during the first two seasons of life. Effectiveness during second season was low or null, although it may be underestimated due to unavoidable survivor bias. The RSV hospitalisation rate among immunised children did not rebound in the second season.

**Key public health message**

**What did you want to address in this study and why?**

Universal immunisation with nirsevimab to prevent severe RSV in children born or entering their first respiratory virus season was first implemented in four countries between October 2023 and March 2024. Such unprecedented intervention needs real-world data to confirm its effectiveness in the long term and to understand its overall effect on the burden and epidemiology of RSV in young children.

**What have we learnt from this study?**

Nirsevimab immunisation reduced RSV hospital admissions by 64% to 74% during the first two RSV seasons of life overall. Most of this benefit was due to the protection during the first season, when children are at higher risk and have been recently immunised, while its benefit during the second season was low or null.

**What are the implications of your findings for public health?**

The net benefit of nirsevimab immunisation across the first two seasons of life was high, with no shift in the burden of RSV to the second post-immunisation season, endorsing current recommendations. Some residual benefit may extend to the second season for children born and immunised in February and March, which could be factored in when considering to include these birth cohorts in the immunisation programme.

## Introduction

Bronchiolitis or pneumonia caused by respiratory syncytial virus (RSV) infection is one of the leading causes of hospital admission in young children [1,2]. It is estimated that RSV caused 33 million infections, 3.6 million hospitalisations and over 100,000 deaths globally in children younger than 5 years in 2019 [3]. In temperate climates, incidence concentrates during epidemic periods, typically between October and March in the northern hemisphere, with over 25% of infants undergoing an RSV infection and 1.8% of infants requiring hospitalisation during their first RSV season [2]. Major risk factors for severe clinical course apart from younger age include prematurity, cardiopulmonary disease and other comorbidities [1,2,4]. However, most children hospitalised with RSV infection are previously healthy [1,4].

Nirsevimab, a monoclonal antibody against the RSV pre-fusion protein, was approved by the European Medicines Agency in October 2022 and by the United States Food and Drug Administration in July 2023 for the prevention of severe RSV infection in children born or entering their first respiratory season. A single intramuscular dose of 50 mg is recommended for infants weighing less than 5 kg and one of 100 mg for those weighing 5 kg or more [5]. Owing to its extended half-life of ca 70 days [6,7], its efficacy of 77–83% in preventing RSV hospitalisation up to 150 days post immunisation [8-10], its lower price compared with previously available anti-RSV monoclonal antibodies, and the favourable safety profile [11], in autumn 2023 Spain recommended a single dose of nirsevimab to all children born 1 April 2023 through 31 March 2024 [12], becoming one of the four countries worldwide to first implement this population-wide preventive immunisation. Acceptability was very high, reaching a coverage of around 90% [13].

Post-authorisation observational studies have confirmed very high effectiveness and impact of first-season nirsevimab immunisation under real-life conditions for diverse population groups [14-20]. However, the long-term protection of this immunisation remains unclear. Neutralising RSV antibody levels were observed to decline progressively from >140-fold higher than at-birth maternal levels 1 month after nirsevimab administration to just >7-fold higher 1 year later [21], and it is uncertain whether these residual antibody levels in children over 1 year of age result in any clinical protection. On the other hand, prevention of RSV infections during the first season could shift the burden of disease to the second season, which could decrease the net benefit of immunisation programmes [22].

This study aimed to estimate the effectiveness of administering a single nirsevimab dose to healthy term-born infants before their first RSV season in preventing RSV hospitalisation throughout the first two seasons of life. We also estimated specific effectiveness during the first and second season, as well as for different population groups and case characteristics.

## Methods

### Study population, design, and eligibility criteria

We conducted a nested case–control study within the underlying cohort of children born between 1 April 2023 and 31 March 2024 in public hospitals in 16 of the 19 autonomous regions of Spain. Thirteen regions participated with the whole public hospital network (the main healthcare provider in Spain) in the entire region or in selected highly populated provinces or islands, while three regions included only some public hospitals with better data accessibility. The cohort included 36% of all births in Spain between April 2023 and March 2024.

Cases were children first admitted to one of the participating hospitals for lower respiratory tract infection, apnoea, or sepsis from the onset of the 2023/24 nirsevimab immunisation campaign in each Spanish region (mostly between 25 September and 6 October 2023) until 31 March 2024 (first post-immunisation RSV season) or from 1 October 2024 until 31 March 2025 (second post-immunisation RSV season), who were PCR-positive for RSV 10 days before to 3 days after the hospitalisation date. Cases hospitalised in the inter-season period (1 April to 30 September 2024) were not eligible.

We included all eligible cases in the source population during the first RSV season, which were already covered in a previous publication on first-season effectiveness [19]. However, during the second RSV season, due to logistic constraints (inability to collect complete information from all eligible cases and their matched controls within the planned timeframe in populated regions with a large number of cases), we selected only a region-stratified random sample of about two-thirds of all eligible cases; in Supplementary Table S1, we append the distribution of eligible and selected cases by autonomous region. By 31 January 2025, all regions had enough eligible cases, and a random sample of cases was selected from those eligible up to that date. Thus, none of the 98 subsequent cases in February and March 2025 (12.1% of all 808 eligible cases in the second season) were included in the study.

For each case, we selected a density (risk-set) sample of four controls matched to the case on province and date of birth (± 2 days or exceptionally ± 4 days in two small regions) among children in the source population who had not moved out of the region, died, or been hospitalised for RSV infection up to the hospitalisation date of the case. The matching date of controls was the hospitalisation date of their index case, thus achieving case–control matching on both calendar time and age. Controls were identified from birth registries or, in regions with no access to birth registries in real time, from population-based registries of the newborn screening programme for metabolic disorders, which is universally performed at birth in Spain. Where birth time was available, the two controls born immediately before and after the case were selected; otherwise, controls were selected randomly from those born within 2 days.

Matched sets for cases born between 1 April 2023 and the start of the 2023/24 immunisation campaign in each region constituted the catch-up immunisation study, whereas matched sets for cases born between the 2023/24 campaign onset and 31 March 2024 formed the at-birth immunisation study. We excluded four regions from the catch-up study because their 2023/24 campaigns began in late October or November or were only implemented for high-risk children. Since cases during the second RSV season were sampled with different selection probabilities by region, matched sets in the catch-up and at-birth immunisation studies were assigned sampling weights inversely proportional to the sampling fraction of cases within each RSV season and region to restore the distribution of cases in the underlying birth cohorts. The assigned sampling weights by study, RSV season and region are appended in Supplementary Table S1.

Authorised personnel in each region collected and curated data from clinical records through manual extraction, except in three regions that relied on automated hospital databases. Data were gathered into a RedCap data collection form or submitted in CSV format using common metadata, with an anonymised code that prevented individual identification. Test results and immunisation information were obtained from registries through manual query or automated extraction. All information on cases and controls was collected up to the matching date.

### Data analysis

We used target trial emulation, an approach to observational data analysis within the causal framework [23]. To this end, we first specified the randomised trial that would answer the causal question (target trial) and then emulated it from our observational data. The target trial would aim to evaluate the long-term effectiveness of a single nirsevimab dose in preventing RSV hospitalisation during the first two RSV seasons in healthy term-born children. The randomly assigned intervention would be the administration of a single nirsevimab dose in the first 30 days of the 2023/24 campaign for catch-up immunisation or in the first 14 days of life for at-birth immunisation, allowing for a so-called grace period for nirsevimab administration. Intention-to-treat (ITT) and per-protocol (PP) effects would be estimated.

For the emulation of this hypothetical trial, we first excluded from the eligible population high-risk children (105/1,190 cases and 177/4,758 controls) born before 35 weeks of gestation or with previous comorbidities, including congenital heart disease, bronchopulmonary dysplasia, cardiopulmonary bypass, immunodeficiency, cystic fibrosis, congenital metabolic disorders, neuromuscular disorders and Down syndrome. We used our matched density case–control sample to specify the underlying cohort from which cases and controls were selected, in which we implemented cloning and censoring to avoid immortal time bias [19,24].

We created two clones for each participant, assigned one clone to immunisation and the other to no immunisation, and censored them when they deviated from the assigned immunisation group. Clones in the immunisation group were censored at the end of the intervention grace period (day 30 of 2023/24 campaign or day 14 of life) if they reached that time without receiving nirsevimab, and clones in the non-immunisation group were censored at any time they received nirsevimab during the grace period. For PP analysis, clones in both groups were also censored at any later time they received out-of-protocol nirsevimab during the 2023/24 and 2024/25 RSV seasons, that is, when they received any nirsevimab dose after the end of the grace period, including any dose administered during the second season. Note that for both ITT and PP analyses, only one clone of cases and controls hospitalised or matched after the grace period remained under follow-up beyond the end of that period: the clone whose observed immunisation status at the end of the grace period conformed to the assigned intervention group. However, clones of cases and controls hospitalised or matched during the grace period without having been immunised were counted in both intervention groups, thus correcting the immortal time bias of standard observational analyses (immunised children must remain free from hospitalisation until nirsevimab administration) [24].

Clones of the originally selected cases and their matched controls who were not censored before the matching date constituted a density case–control sample from the censored follow-up of the underlying cloned cohort [19]. Thus, causal ITT and PP effectiveness were estimated as 1 minus the hospitalisation rate ratios from conditional logistic models on the immunisation assigned to uncensored clones of cases and controls.

Given the different selection probabilities of cases and controls and the potential for informative censoring of their clones (non-random immunisation in the population), conditional logistic models were weighted by the product of sampling and censoring weights [25]. Stabilised censoring weights were calculated as the probability of cloned cases and controls of remaining uncensored at the matching date given their assigned immunisation and baseline factors, including sex (female or male), gestational age (35–36, 37–38 or ≥ 39 weeks), birthweight (< 2,500, 2,500–3,000 or ≥ 3,000 g), and multiple pregnancy (no or yes), divided by the same probability further conditional on previous non-RSV hospitalisation [26]. These probabilities were estimated using sampling-weighted pooled logistic models of the daily immunisation history among controls, as described in the Supplement. Since censoring weights corrected the informative censoring of clones due to prior non-RSV hospitalisation within levels of the baseline factors, sampling- and censoring-weighted conditional logistic models were adjusted for baseline factors. For the effective control for prior non-RSV hospitalisation through weighting see Supplementary Tables S2 and S3. Conservative confidence intervals (CIs) based on robust standard errors were used to account for the correlation induced by cloning and weighting [26].

For comparison, we also obtained pragmatic estimates of effectiveness by naively comparing the actual immunisation status of cases and controls at the matching date and adjusting for the above factors. Sensitivity analyses were conducted by excluding matched sets for cases with co-detection of other respiratory pathogens (analysed by multiplex PCR according to the hospitals’ protocols). Subgroup analyses by baseline factors and case characteristics (invasive or non-invasive mechanical ventilation, intensive care unit (ICU) admission, and RSV subgroup) were performed by including interaction terms of immunisation with baseline factors and stratifying matched sets by case characteristic.

## Results

### Participant characteristics

**Figure 1:**
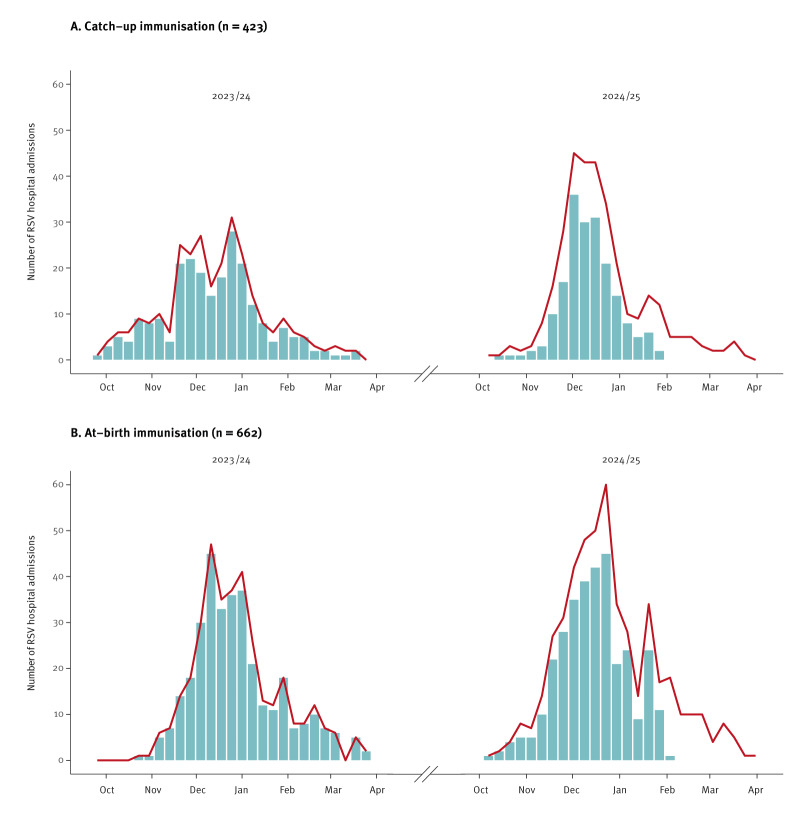
Hospital admissions for respiratory syncytial virus infection among children born before and after the start of the 2023/24 nirsevimab immunisation campaign, Spain, October 2023–March 2025 (n = 1,085)

In the catch-up immunisation study, we initially recruited all 276 eligible cases hospitalised during the 2023/24 season and 212 of all 320 eligible cases hospitalised during the 2024/25 season. In the at-birth immunisation study, we enrolled all 354 eligible cases during the 2023/24 season and 348 of all 488 eligible cases during the 2024/25 season (Figure 1). After selecting children born at ≥ 35 gestation weeks without major comorbidities, 235 cases and 905 controls for catch-up and 334 cases and 1,292 controls for at-birth immunisation were included from the first season, and 188 cases and 713 controls for catch-up and 328 cases and 1,269 controls for at-birth immunisation were included from the second season. The sampling-weighted proportion of cases occurred during the second season was 54% in catch-up and 58% in at-birth study.

RSV: respiratory syncytial virus.

Data are shown by 2023/24 and 2024/25 epidemiological week. Panel A includes data for children born between 1 April 2023 and the start of the 2023/24 nirsevimab immunisation campaign. Panel B includes data for children born between the 2023/24 campaign onset and 31 March 2024. Lines represent all eligible hospital admissions in the source population among both healthy term-born and high-risk children, and bars correspond to cases included in the study among healthy term-born children.

The positive PCR test for RSV was performed on the day of hospital admission in 72% of cases and at most 1 day before or after admission in 92% of cases. The RSV subgroup was missing for 74% of cases. During the second season, the severity of cases, defined by need of mechanical ventilation (invasive or non-invasive) and ICU admission, was lower in both studies and the proportion with co-detection of other respiratory pathogens was higher. Mean time from immunisation to matching was lower for children immunised at birth (223 days vs 266 days in children immunised as catch-up), as well as for cases that occurred during the first season and their controls (68–74 days in the catch-up study and 45–46 days in the at-birth study during the first season vs 429 and 352–353 days during the second season, respectively). In the at-birth immunisation study, mean time from immunisation to matching increased gradually across birth cohorts (182, 248 and 286 days for children born in October–November 2023, December 2023–January 2024 and February–March 2024, respectively). Characteristics of cases and controls are shown in Table 1 and Table 2. The distribution of cases and controls by autonomous region is appended in Supplementary Tables S4 and S5.

**Table 1:** Characteristics of cases hospitalised for respiratory syncytial virus infection and density-matched population controls among healthy term-born children in the catch-up nirsevimab immunisation study, Spain, October 2023–March 2025 (n = 2,041)

Characteristic	2023/24 RSV season				2024/25 RSV season				Both RSV seasons			
	Cases	%	Controls	%	Cases	%	Controls	%	Cases	%	Controls	%
Number of participants	235		905		188		713		423		1,618	
Month of birth												
April 2023	16	6.8	62	6.9	21	10.9	81	11.1	37	9.0	143	9.1
May 2023	24	10.2	93	10.3	33	16.5	130	17.1	57	13.6	223	13.9
June 2023	32	13.6	122	13.5	26	14.6	98	14.4	58	14.1	220	14.0
July 2023	46	19.6	177	19.6	22	12.0	80	11.6	68	15.5	257	15.3
August 2023	44	18.7	171	18.9	43	23.7	162	23.9	87	21.4	333	21.6
September 2023	68	28.9	261	28.8	40	21.1	151	20.8	108	24.7	412	24.5
October 2023	5	2.1	19	2.1	3	1.2	11	1.1	8	1.6	30	1.6
Mean age in days at hospital admission (SD)	143 (62)		NA		522 (54)		NA		349 (198)		NA	
Sex												
Female	100	42.6	424	46.9	91	47.3	335	45.7	191	45.1	759	46.2
Male	135	57.4	481	53.1	97	52.7	378	54.3	232	54.9	859	53.8
Gestational age (weeks)												
35–36	15	6.4	46	5.1	7	3.6	18	2.4	22	4.9	64	3.6
37–38	61	26.0	215	23.8	63	31.4	164	22.5	124	28.9	379	23.1
≥ 39	159	67.7	644	71.2	118	65.0	531	75.1	277	66.2	1,175	73.3
Birthweight (g)												
< 2,500	15	6.4	45	5.0	12	7.3	33	4.7	27	6.9	78	4.8
2,500–3,000	40	17.0	201	22.2	49	25.8	161	22.8	89	21.8	362	22.5
≥ 3,000	180	76.6	659	72.8	127	66.8	519	72.4	307	71.3	1,178	72.6
Multiple pregnancy												
No	232	98.7	896	99.2	184	97.7	704	98.4	416	98.2	1,600	98.8
Yes	3	1.3	7	0.8	4	2.3	9	1.6	7	1.8	16	1.2
Unknown	0	NA	2	NA	0	NA	0	NA	0	NA	2	NA
Previous non-RSV hospitalisation												
No	201	85.5	808	89.3	147	76.6	656	92.1	348	80.7	1,464	90.8
Yes	34	14.5	97	10.7	41	23.4	57	7.9	75	19.3	154	9.2
Co-detection of other respiratory pathogens^a^												
Any pathogen	45	19.1	NA		61	36.1	NA		106	28.4	NA	
Adenovirus	1	0.4			28	17.4			29	9.7		
Bacteria	4	1.7			4	3.0			8	2.4		
Bocavirus	2	0.9			4	2.1			6	1.5		
Influenza virus	2	0.9			1	0.9			3	0.9		
Metapneumovirus	1	0.4			0	0.0			1	0.2		
Non-SARS-CoV-2 coronavirus	1	0.4			5	3.3			6	2.0		
Parainfluenza virus	3	1.3			3	1.8			6	1.6		
Rhinovirus/enterovirus	26	11.1			40	22.1			66	17.1		
SARS-CoV-2	4	1.7			1	0.9			5	1.3		
Unspecified	6	2.6			0	0.0			6	1.2		
Case severity^a^												
Mechanical ventilation: all	84	35.7	NA		50	28.6	NA		134	31.9	NA	
Mechanical ventilation: invasive	4	1.7			1	0.9			5	1.3		
Mechanical ventilation: non-invasive	80	34.0			49	27.7			129	30.6		
ICU admission	32	13.6			15	8.1			47	10.6		
Deceased	0	0.0			0	0.0			0	0.0		
RSV subgroup												
A	31	75.6	NA		44	64.1	NA		75	67.7	NA	
B	10	24.4			23	35.9			33	32.3		
Unknown	194	NA			121	NA			315	NA		
Nirsevimab immunisation^b^												
Per protocol	117	49.8	689	76.1	158	84.0	575	79.0	275	68.4	1,264	77.7
Out of protocol	14	6.0	88	9.7	15	8.7	88	14.0	29	7.5	176	12.0
Not immunised	104	44.3	128	14.1	15	7.3	50	7.0	119	24.2	178	10.3
Mean time in days from immunisation to matching^c^ (SD)	74 (32)		68 (33)		429 (19)		429 (19)		311 (170)		266 (182)	
Multiple immunisation^d^												
No	235	100	905	100	188	100	712	99.9	423	100	1,617	99.9
Yes	0	0.0	0	0.0	0	0.0	1	0.1	0	0.0	1	0.1

ICU: intensive care unit; NA: not applicable; RSV: respiratory syncytial virus; SARS-CoV-2: severe acute respiratory syndrome coronavirus 2; SD: standard deviation.

^a^ Not mutually exclusive categories.

^b^ Nirsevimab immunisation before the matching date. The per-protocol immunisation period was the first 30 days of 2023/24 campaign.

^c^ Time from immunisation to matching among per-protocol immunised children.

^d^ Immunisation with a second dose of nirsevimab during the 2024/25 RSV season.

Data are unweighted counts and sampling-weighted percentages, except sampling-weighted means (sampling-weighted standard deviations) for age at hospital admission and time from immunisation to matching.

**Table 2:** Characteristics of cases hospitalised for respiratory syncytial virus infection and density-matched population controls among healthy term-born children in the at-birth nirsevimab immunisation study, Spain, October 2023–March 2025 (n = 3,223)

Characteristic	2023/24 RSV season				2024/25 RSV season				Both RSV seasons			
	Cases	%	Controls	%	Cases	%	Controls	%	Cases	%	Controls	%
Number of participants	334		1,292		328		1,269		662		2,561	
Month of birth												
September 2023	5	1.5	20	1.5	2	0.8	8	0.8	7	1.1	28	1.1
October 2023	118	35.3	457	35.4	42	12.5	162	12.4	160	22.1	619	22.1
November 2023	118	35.3	456	35.3	60	18.0	234	18.1	178	25.3	690	25.3
December 2023	70	21.0	272	21.1	73	21.9	282	21.9	143	21.5	554	21.6
January 2024	19	5.7	71	5.5	58	17.7	222	17.6	77	12.7	293	12.5
February 2024	4	1.2	16	1.2	55	16.2	213	16.3	59	9.9	229	9.9
March 2024	0	0.0	0	0.0	38	12.8	148	12.9	38	7.4	148	7.5
Mean age in days at hospital admission (SD)	47 (26)		NA		354 (53)		NA		225 (158)		NA	
Sex												
Female	132	39.5	627	48.5	157	47.6	613	48.3	289	44.2	1,240	48.4
Male	202	60.5	665	51.5	171	52.4	656	51.7	373	55.8	1,321	51.6
Gestational age (weeks)												
35–36	22	6.6	57	4.4	19	5.9	60	4.9	41	6.2	117	4.7
37–38	95	28.4	309	23.9	101	31.8	333	26.4	196	30.4	642	25.3
≥ 39	217	65.0	926	71.7	208	62.3	876	68.8	425	63.4	1,802	70.0
Birthweight (g)												
< 2,500	16	4.8	75	5.8	32	10.4	67	5.3	48	8.0	142	5.5
2,500–3,000	67	20.1	264	20.4	73	22.1	248	19.8	140	21.2	512	20.1
≥ 3,000	251	75.1	952	73.7	223	67.5	954	74.9	474	70.7	1,906	74.4
Unknown	0	NA	1	NA	0	NA	0	NA	0	NA	1	NA
Multiple pregnancy												
No	323	96.7	1,266	98.4	310	94.5	1,233	97.1	633	95.4	2,499	97.6
Yes	11	3.3	21	1.6	18	5.5	36	2.9	29	4.6	57	2.4
Unknown	0	NA	5	NA	0	NA	0	NA	0	NA	5	NA
Previous non-RSV hospitalisation												
No	291	87.1	1,130	87.5	274	82.7	1,140	89.6	565	84.6	2,270	88.7
Yes	43	12.9	162	12.5	54	17.3	129	10.4	97	15.4	291	11.3
Co-detection of other respiratory pathogens^a^												
Any pathogen	59	17.7	NA		126	39.6	NA		185	30.4	NA	
Adenovirus	2	0.6			50	15.9			52	9.5		
Bacteria	4	1.2			7	2.6			11	2.0		
Bocavirus	0	0.0			15	4.3			15	2.5		
Influenza virus	4	1.2			8	2.6			12	2.0		
Metapneumovirus	0	0.0			1	0.3			1	0.1		
Non-SARS-CoV-2 coronavirus	6	1.8			14	4.1			20	3.2		
Parainfluenza virus	1	0.3			9	3.0			10	1.8		
Rhinovirus/enterovirus	33	9.9			73	22.1			106	16.9		
SARS-CoV-2	5	1.5			2	0.6			7	1.0		
Unspecified	9	2.7			0	0.0			9	1.1		
Case severity^a^												
Mechanical ventilation: all	114	34.1	NA		90	27.8	NA		204	30.4	NA	
Mechanical ventilation: invasive	6	1.8			5	1.5			11	1.6		
Mechanical ventilation: non-invasive	108	32.3			85	26.2			193	28.8		
ICU admission	60	18.0			28	7.9			88	12.1		
Deceased	1	0.3			0	0.0			1	0.1		
RSV subgroup												
A	36	58.1	NA		88	81.7	NA		124	74.5	NA	
B	26	41.9			20	18.3			46	25.5		
Unknown	272	NA			220	NA			492	NA		
Nirsevimab immunisation^b^												
Per protocol	231	69.2	1,170	90.6	291	89.8	1,146	90.7	522	81.1	2,316	90.7
Out of protocol	8	2.4	51	3.9	3	0.9	25	2.0	11	1.5	76	2.8
Not immunised	95	28.4	71	5.5	34	9.3	98	7.2	129	17.4	169	6.5
Mean time in days from immunisation to matching^c^ (SD)	46 (26)		45 (26)		353 (51)		352 (52)		243 (154)		223 (158)	
Multiple immunisation^d^												
No	334	100	1,292	100	327	99.8	1,262	99.5	661	99.9	2,554	99.7
Yes	0	0.0	0	0.0	1	0.2	7	0.5	1	0.1	7	0.3

ICU: intensive care unit; NA: not applicable; RSV: respiratory syncytial virus; SARS-CoV-2: severe acute respiratory syndrome coronavirus 2; SD: standard deviation.

^a^ Not mutually exclusive categories.

^b^ Nirsevimab immunisation before the matching date. The per-protocol immunisation period was the first 14 days of life.

^c^ Time from immunisation to matching among per-protocol immunised children.

^d^ Immunisation with a second dose of nirsevimab during the 2024/25 RSV season.

Data are unweighted counts and sampling-weighted percentages, except sampling-weighted means (sampling-weighted standard deviations) for age at hospital admission and time from immunisation to matching.

### Effectiveness of nirsevimab over two seasons

**Figure 2:**
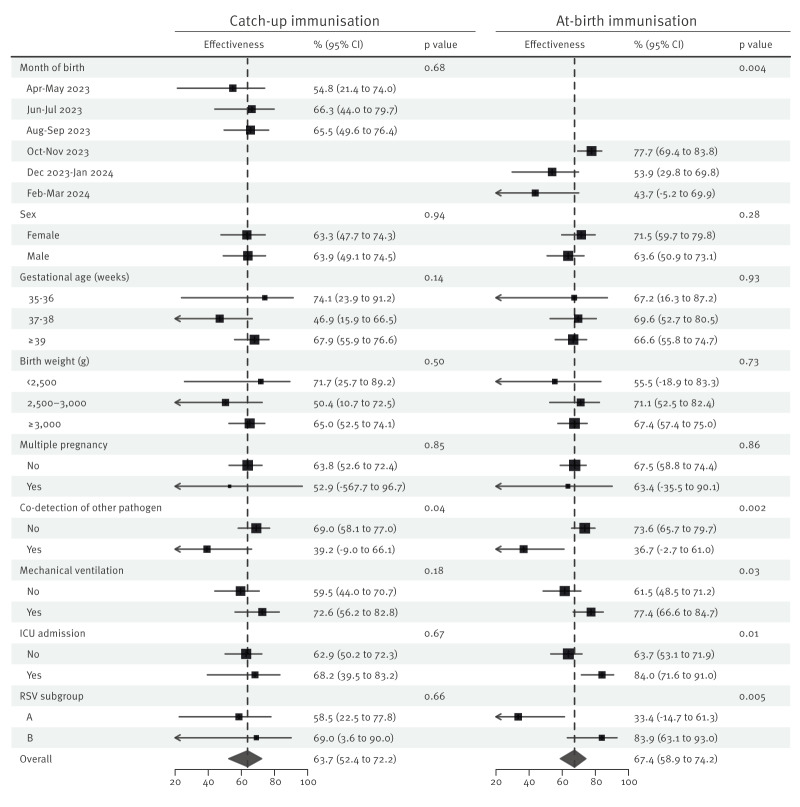
Per-protocol effectiveness of nirsevimab immunisation against hospitalisation for respiratory syncytial virus infection during both 2023/24 and 2024/25 seasons among healthy term-born children, Spain, October 2023–March 2025 (n= 5,034 clones)

CI: confidence interval; ICU, intensive care unit; RSV: respiratory syncytial virus.

Nirsevimab administered as catch-up decreased the rate of RSV hospitalisation during the first two RSV seasons by 64% (95% CI: 52–72) according to the PP estimate (Table 3). Effectiveness increased to 69% (95% CI: 58–77) among cases in which RSV was the only respiratory pathogen detected. Catch-up immunisation showed similar, but rather imprecise two-season effectiveness by baseline children characteristics, case severity or RSV subgroup (Figure 2).

**Table 3:** Two-season effectiveness of catch-up nirsevimab immunisation against hospitalisation for respiratory syncytial virus infection among healthy term-born children, Spain, October 2023–March 2025 (based on data from n = 2,039 children)

	2023/24 RSV season						2024/25 RSV season						Both RSV seasons					
	Cases		Controls		Effectiveness		Cases		Controls		Effectiveness		Cases		Controls		Effectiveness	
	Immunised	Total	Immunised	Total	%	95% CI	Immunised	Total	Immunised	Total	%	95% CI	Immunised	Total	Immunised	Total	%	95% CI
Overall																		
Pragmatic^a^	131	235	775	903	85.1	79.2 to 89.4	173	188	663	713	12.8	−59.2 to 52.2	304	423	1,438	1,616	72.3	63.0 to 79.3
Intention to treat^b^	137	255	737	953	65.8	55.8 to 73.6	158	188	575	713	−43.1	−117 to 5.7	295	443	1,312	1,666	37.6	22.3 to 50.0
Per protocol^c^	137	241	737	865	77.6	69.6 to 83.5	158	173	575	625	−7.7	−87.9 to 38.3	295	414	1,312	1,490	63.7	52.4 to 72.2
Single RSV infection^d^																		
Pragmatic^a^	104	190	627	730	84.8	78.1 to 89.5	115	127	455	484	34.1	−36.8 to 68.2	219	317	1,082	1,214	76.6	67.3 to 83.2
Intention to treat^b^	109	206	591	768	65.3	53.8 to 73.9	105	127	402	484	−5.2	−70.4 to 35.1	214	333	993	1,252	47.5	33.0 to 58.9
Per protocol^c^	109	195	591	694	77.4	68.4 to 83.8	105	117	402	431	25.5	−44.6 to 61.6	214	312	993	1,125	69.0	58.1 to 77.0

CI: confidence interval; RSV: respiratory syncytial virus.

^a^ Pragmatic estimates of effectiveness (95% CIs) were obtained from sampling-weighted conditional logistic models based on the actual immunisation status of cases and controls at the matching date and adjusting for sex, gestational age, birthweight, multiple pregnancy, and previous non-RSV hospitalisation up to that date.

^b^ Causal estimates of intention-to-treat effectiveness (95% CIs) were obtained from sampling- and censoring-weighted conditional logistic models based on the assigned immunisation among uncensored clones of cases and controls at the end of the intervention grace period (day 30 of 2023/24 campaign). The increase in the number of cases and controls (20 cases and 50 controls for overall analysis in both RSV seasons) corresponded to clones who remained uncensored in both immunisation groups.

^c^ Causal estimates of per-protocol effectiveness (95% CIs) were obtained from sampling- and censoring-weighted conditional logistic models based on the assigned immunisation among uncensored clones of cases and controls at the matching date. The decrease in the number of cases and controls (29 cases and 176 controls for overall analysis in both RSV seasons) corresponded to clones who received out-of-protocol nirsevimab after the intervention grace period.

^d^ Excluding 106 cases with co-detection of other respiratory pathogen and their matched 403 controls.

Analyses were based on cases and controls with complete covariate information. Conservative 95% CIs were calculated using robust standard errors.

The area of each square is inversely proportional to the variance. Horizontal lines represent 95% CIs.

At-birth nirsevimab immunisation reduced the rate of RSV hospitalisation during two seasons by 67% (95% CI: 59–74) according to the PP estimate (Table 4). Effectiveness increased to 74% (95% CI: 66–80) when restricted to cases where RSV was the only respiratory pathogen detected. Effectiveness of at-birth immunisation decreased progressively from 78% (95% CI: 69–84) in children born in October and November 2023 to 54% (95% CI: 30–70) in those born in December 2023 and January 2024 and to 44% (95% CI: −5 to 70) in those born in February and March 2024. It was higher for cases requiring mechanical ventilation or ICU admission and for infections caused by RSV B (Figure 2). 

**Table 4:** Two-season effectiveness of at-birth nirsevimab immunisation against hospitalisation for respiratory syncytial virus infection among healthy term-born children, Spain, October 2023–March 2025 (based on data from n = 3,217 children)

	2023/24 RSV season						2024/25 RSV season						Both RSV seasons					
	Cases		Controls		Effectiveness		Cases		Controls		Effectiveness		Cases		Controls		Effectiveness	
	Immunised	Total	Immunised	Total	%	95% CI	Immunised	Total	Immunised	Total	%	95% CI	Immunised	Total	Immunised	Total	%	95% CI
Overall																		
Pragmatic^a^	239	334	1,215	1,286	86.6	81.4 to 90.4	294	328	1,171	1,269	25.7	−9.6 to 49.6	533	662	2,386	2,555	69.9	61.8 to 76.2
Intention to treat^b^	236	339	1,167	1,288	79.2	72.6 to 84.3	291	328	1,146	1,269	1.4	−46.5 to 33.7	527	667	2,313	2,557	59.0	48.8 to 67.1
Per protocol^c^	236	331	1,167	1,238	84.3	78.7 to 88.5	290	324	1,139	1,237	19.5	−20.7 to 46.3	526	655	2,306	2,475	67.4	58.9 to 74.2
Single RSV infection^d^																		
Pragmatic^a^	193	275	998	1,060	86.3	80.6 to 90.3	179	202	727	780	41.9	7.5 to 63.6	372	477	1,725	1,840	75.3	67.7 to 81.1
Intention to treat^b^	191	280	960	1,062	79.8	72.9 to 85.0	176	202	706	780	19.9	−29.3 to 50.3	367	482	1,666	1,842	66.1	56.5 to 73.5
Per protocol^c^	191	273	960	1,022	84.1	78.1 to 88.5	175	198	702	755	37.4	−2.5 to 61.8	366	471	1,662	1,777	73.6	65.7 to 79.7

CI: confidence interval; RSV: respiratory syncytial virus.

^a^ Pragmatic estimates of effectiveness (95% CIs) were obtained from sampling-weighted conditional logistic models based on the actual immunisation status of cases and controls at the matching date and adjusting for sex, gestational age, birthweight, multiple pregnancy, and previous non-RSV hospitalisation up to that date.

^b^ Causal estimates of intention-to-treat effectiveness (95% CIs) were obtained from sampling- and censoring-weighted conditional logistic models based on the assigned immunisation among uncensored clones of cases and controls at the end of the intervention grace period (day 14 of life). The increase in the number of cases and controls (five cases and two controls for overall analysis in both RSV seasons) corresponded to clones who remained uncensored in both immunisation groups.

^c^ Causal estimates of per-protocol effectiveness (95% CIs) were obtained from sampling- and censoring-weighted conditional logistic models based on the assigned immunisation among uncensored clones of cases and controls at the matching date. The decrease in the number of cases and controls (12 cases and 82 controls for overall analysis in both RSV seasons) corresponded to clones who received out-of-protocol nirsevimab after the intervention grace period.

^d^ Excluding 185 cases with co-detection of other respiratory pathogen and their matched 716 controls.

Analyses were based on cases and controls with complete covariate information. Conservative 95% CIs were calculated using robust standard errors.

Intention-to-treat analyses severely underestimated effectiveness in both studies, while pragmatic analyses overestimated it (Table 3, Table 4).

### Specific effectiveness of nirsevimab during the first and second seasons

The PP effectiveness was very high during the first post-immunisation season, reaching 78% (95% CI: 70–84) for catch-up immunisation and 84% (95% CI: 79–88) for at-birth immunisation, with identical results among cases with single RSV infection (Table 3, Table 4). In contrast, we found low PP effectiveness during the second post-immunisation season, ranging from −8% to 20% overall and from 26% to 37% for single RSV infections, mostly compatible with null effectiveness (Table 3, Table 4).

## Discussion

Single-dose nirsevimab immunisation in healthy term-born children born or entering their first RSV season reduced the rate of RSV hospital admissions throughout the first two seasons of life by 64–74%. Two-season effectiveness was similar for children immunised at birth and those immunised as catch-up, but tended to be higher for single RSV infections (without co-detection of other respiratory pathogen). Most of the estimated benefit was attributable to nirsevimab effectiveness during the first season (77–84%), while its effectiveness during the second season was low or null (−8% to 37%). The effectiveness over the first two seasons of life estimated the net benefit of nirsevimab immunisation during this most-at-risk period for severe disease. If the risk of infection rebounded in the second season, it could (hypothetically) outweigh or greatly reduce the benefit observed during the first season, something that our results do not show.

Protection against severe RSV disease in the youngest children is a priority for nirsevimab immunisation programmes, as they bear the highest disease burden [2,3]. A previous analysis of the birth cohorts represented in our study showed that the risk of RSV hospitalisation in non-immunised newborns during their first 2023/24 season was 3% overall and reached 7% in those born 1 month before the RSV epidemic peak [20], consistent with cohort studies [1,2,4]. High effectiveness shortly after nirsevimab administration is concordant with efficacy of 77–83% from randomised clinical trials in late pre-term or term-born children up to 150 or 180 days of follow-up [9,27].

In contrast, the risk of severe RSV is substantially lower in the second year of life [1,3] and so is the number of RSV hospitalisations potentially preventable by immunisation. Interestingly, in the birth cohorts included in our study, the rate of RSV hospitalisation was slightly higher in the second RSV season compared with the first season, possibly due to the very high effectiveness of nirsevimab in the first season, though lower bed occupancy due to the impact of nirsevimab could increase admission of less severe cases. We found fewer severe cases requiring mechanical ventilation or ICU admission in the second season.

The lower or null protection during the second post-immunisation season was expected and is concordant with the reduced neutralising RSV antibody titres remaining 1 year after nirsevimab immunisation [21]. Similar risks of RSV hospital admission of 0.2% and 0.3% during the second RSV season were also found for nirsevimab and placebo groups, respectively, in the extended passive follow-up of the MELODY trial [22]. However, we cannot rule out in either study that some low-level nirsevimab protection may extend to the second season, since period-specific effect estimates at any post-baseline interval are prone to selection bias due to a higher depletion of susceptible children in the non-immunised group during previous intervals, which underestimates subsequent effectiveness [28]. In contrast, available evidence strongly indicates that the risk of RSV hospitalisation does not rebound in the second RSV season, probably because nirsevimab does not result in sterilising immunity nor prevents an active immune response [21]. Therefore, RSV infection in immunised children would be subclinical but would still generate immune memory that would protect them in future contacts with the virus.

Nirsevimab effectiveness was higher in preventing more severe outcomes, such as need of mechanical respiratory support (invasive or non-invasive) or ICU admission, this higher effectiveness being more evident in the at-birth immunisation cohort. These outcomes overcome the heterogeneity in severity among hospitalised cases, which can vary depending on the hospital, age of the child, and bed occupancy. The findings are in agreement with randomised controlled trials and observational studies, which have found similar or higher effectiveness for very severe RSV infections [9,10,29]. A recent study found that the clinical presentation among 34 children immunised with nirsevimab more than 6 months earlier was similar to that of non-immunised children, supporting no rebound in case severity [30].

Effectiveness was higher for single RSV infections. About 30% of RSV hospitalisations in our study had co-detection of other pathogens, consistent with common co-infection in other case series [2,31]. Differences in clinical presentation have been described for distinct RSV and non-RSV virus combinations, some of them associated, though not consistently, with RSV case severity [31-33]. Since nirsevimab does not confer protection against non-RSV viruses [32], it is possible that the co-detected virus among breakthrough RSV cases plays a relevant role in disease severity, decreasing the preventive potential of nirsevimab and its estimated effectiveness, although this hypothesis needs further investigation. The larger proportion of co-detections in our study during the second season, when RSV infection was itself less severe, would support this hypothesis.

We unexpectedly found a higher effectiveness for RSV B vs A in children immunised at birth [8], which may result from bias, as RSV subgroup was missing for 74% of our cases. Nirsevimab targets a highly conserved antigen site of the RSV pre-fusion protein of both viral subgroups [7,34], with less than 1% of RSV B strains showing reduced susceptibility to nirsevimab [34]. On the other hand, RSV B may exhibit substitutions that increase susceptibility, which merits further research [35].

Little or no differences were found between population groups, except for month of birth. Effectiveness decreased from 78% to 54% and 44% in children born in October and November, December and January, and February and March of the 2023/24 RSV season, respectively. This gradual decline in effectiveness across birth cohorts was probably due to the increasing proportion of cases occurring during their second season (37%, 67% and 97%, respectively) at an average of 182, 248 and 286 days after nirsevimab immunisation. The reduced, but still substantial effectiveness for infants born at the end of the season, when RSV circulation was already low, suggests that certain protection extends to the following season and supports ongoing inclusion of these birth cohorts into the immunisation programmes.

Our study has some limitations. Those related to the case–control study design and potential residual confounding have been previously discussed [19]. In this study over two seasons, to avoid bias due to lower sampling fraction of cases in the second season, we weighted selected cases to represent the underlying population incidence in each season. However, the overall estimate of effectiveness may vary if relative RSV circulation across the two seasons varies. Moreover, since case recruitment during the second season was truncated on 31 January 2025, effectiveness in the second season might be overestimated compared with a study that included cases occurring in February and March, when children are older and longer time has elapsed since immunisation. However, only 12% of cases in the second season occurred in these later months. In addition, we cannot rule out some residual confounding by previous non-severe RSV infection in the estimated two-season effectiveness, as RSV is not tested for in routine primary care practice. Finally, our findings cannot be extrapolated to infants born very prematurely (below 35 weeks of gestation) or diagnosed with comorbidities, who were excluded.

## Conclusion

We observed a large benefit of nirsevimab immunisation in healthy term-born children during the first two seasons of life overall, mostly attributable to its effectiveness during the first season. No rebound in RSV hospitalisation rate was observed during the second season, where low or null residual benefit of nirsevimab immunisation was found, albeit possibly underestimated due to differential depletion of susceptibles. Our results endorse immunisation with a single nirsevimab dose as an effective intervention to decrease the risk of severe RSV infection in healthy term-born children, although decisions on immunisation programmes also need to be informed by cost-effectiveness evaluations.

## Data Availability

Fully anonymised and non-identifiable data (i.e. only for regions where more than five cases per province are available) can be made available upon reasonable request to the corresponding author, conditioned to agreement of all the investigators from the regions providing the data.

## Supplementary Data

706000 Supplement
