# Supplementary material for: Two-season effectiveness of a single nirsevimab dose against RSV hospitalisation in healthy term-born infants: a population-based case–control study, Spain, October 2023 to March 2025
Source: Euro Surveill. 2026 Mar 5;31(9):2500593. doi: 10.2807/1560-7917.ES.2026.31.9.2500593 (PMC13074286; doi:10.2807/1560-7917.ES.2026.31.9.2500593)
Supplement: Supplement [file 25-00593_MONGE_Supplement.pdf]

**SUPPLEMENTARY MATERIAL**

**Supplement to:** Núñez O, Juaneda J, Martinez-Marcos M, Muñoz Platón E, Rivas Wagner E, Santiago-Pérez M-I, et al. Two-season effectiveness of a single nirsevimab dose against RSV hospitalisation in healthy term-born infants: a population-based case–control study, Spain, October 2023 to March 2025. Euro Surveill. 2026.

This supplementary material is hosted by *Eurosurveillance* as supporting information alongside the above mentioned article, on behalf of the authors, who remain responsible for the accuracy and appropriateness of the content. The same standards for ethics, copyright, attributions and permissions as for the article apply. Supplements are not edited by *Eurosurveillance* and the journal is not responsible for the maintenance of any links or email addresses provided therein.

**Table of contents**

Supplementary methods 2

Supplementary results 6

Supplementary references 8

Supplementary Table S1 9

Supplementary Table S2 10

Supplementary Table S3 11

Supplementary Table S4 12

Supplementary Table S5 13

## Supplementary methods

### *Estimation of censoring weights for intention-to-treat analysis*

To estimate stabilized censoring weights for the intention-to-treat (ITT) analysis of the effectiveness of at-birth nirsevimab immunization during both 2023-24 and 2024-25 respiratory syncytial virus (RSV) seasons, we began by determining the probability that child $j$ in stratum $i$ of the source population without previous hospitalization for RSV infection remained unimmunized with nirsevimab by the end of day $t \geq 0$ of life, first conditional on baseline factors at birth,

$$\pi_{ijt}^{0} = P(X_{ijt} = 0|\mathbf{I}_i, \mathbf{Z}_{ij}, D_{ijt\text{-}1} = 0)$$
$$= \prod_{k=0}^{t} P(X_{ijk} = 0|X_{ijk\text{-}1} = 0, \mathbf{I}_i, \mathbf{Z}_{ij}, D_{ijk\text{-}1} = 0),$$

and then conditional on full factor history up to the previous day $t - 1$,

$$\pi_{ijt}^{1} = P(X_{ijt} = 0|\mathbf{I}_i, \mathbf{Z}_{ij}, \overline{V}_{ijt\text{-}1}, D_{ijt\text{-}1} = 0)$$
$$= \prod_{k=0}^{t} P(X_{ijk} = 0|X_{ijk\text{-}1} = 0, \mathbf{I}_i, \mathbf{Z}_{ij}, \overline{V}_{ijk\text{-}1}, D_{ijk\text{-}1} = 0),$$

where $X_{ijt}$, $D_{ijt}$, and $V_{ijt}$ were indicators of having received nirsevimab, having been hospitalized for RSV infection, and having been hospitalized for other causes by the end of day $t$, respectively, with $X_{ij,\text{-}1} = D_{ij,\text{-}1} = V_{ij,\text{-}1} = 0$ before birth; $\overline{V}_{ijt} = (V_{ij0}, V_{ij1},\ldots, V_{ijt})$ was the history of non-RSV hospitalization up to day $t$; $\mathbf{Z}_{ij}$ were baseline factors, including sex (female or male), gestational age (35–36, 37–38, or ≥39 weeks), birthweight (<2,500, 2,500–3,000, or ≥3,000 g), and multiple pregnancy (no or yes); and $\mathbf{I}_i$ were indicators of population strata for each combination of province and date of birth. Note that, in these conditional probabilities $\pi_{ijt}^{0}$ and $\pi_{ijt}^{1}$ of remaining unimmunized, the factors for days $k$ of life between RSV seasons were by definition equal to 1, as nirsevimab immunization was not possible in this period.

In the ITT analysis of the emulated target trial, cloned children assigned to immunization were censored at day 14 of life if they reached that time without receiving nirsevimab, whereas cloned children assigned to no immunization were censored at any time they received nirsevimab during the first 14 days of life.[1] Thus, in the nested case-control study, child $j$ in population stratum $i$ selected in a matched set for a case hospitalized at day $t$ of life contributed to the ITT analysis with its clone $c = 1$ assigned to immunization who received nirsevimab (or could still have received it) during the first 14 days of life and its clone $c = 0$ assigned to no immunization who did not receive nirsevimab during this initial 14-day period, with stabilized censoring weights

$$W_{ijct}^{C} = \begin{cases} 1, & c = 1,\ 0 \leq t \leq 13, \\ (1 - \pi_{ij13}^{0})/(1 - \pi_{ij13}^{1}), & c = 1,\ t \geq 14, \\ \pi_{ijt}^{0}/\pi_{ijt}^{1}, & c = 0,\ 0 \leq t \leq 13, \\ \pi_{ij13}^{0}/\pi_{ij13}^{1}, & c = 0,\ t \geq 14. \end{cases}$$

The denominator of $W_{ijct}^{C}$ corresponded to the probability of cloned cases and controls remaining uncensored at the matching date given their full factor history. The numerator, which represented the probability of remaining uncensored given only baseline factors, was added to stabilize weights and reduce variance in the weighted estimate of immunization effectiveness.[2]

To estimate the conditional probabilities $\pi_{ijt}^{0}$ and $\pi_{ijt}^{1}$ of being unimmunized in the source population, we used the density sample of controls. Since cases had different selection probabilities, we assigned sampling weights $W_{ij}^{S}$ to cases and their matched controls as the inverse of the sampling fraction of cases within each RSV season and autonomous region (**Supplementary Table S1**).[3] Thus, we estimated the factors in $\pi_{ijt}^{1}$ using a weighted pooled logistic model of the probability that control $j$ in population stratum $i$ remained unimmunized

with nirsevimab at the end of each day $k$ from birth to matching date (excluding the period between RSV seasons when immunization was not possible) among previously unimmunized controls with sampling weights $W_{ij}^{S}$,

$$\text{logit}\{P(X_{ijk} = 0 | X_{ijk\text{-}1} = 0, \mathbf{I}_i, \mathbf{Z}_{ij}, \overline{V}_{ijk\text{-}1}, D_{ijk\text{-}1} = 0)\}$$

$$= \beta_0 + b_p + \boldsymbol{\beta}_1'\mathbf{s}(B_{ij}) + \boldsymbol{\beta}_2'\mathbf{I}(k) + \boldsymbol{\beta}_3'\mathbf{Z}_{ij} + \boldsymbol{\beta}_4'\mathbf{I}(k)V_{ijk\text{-}1}.$$

Due to the sparse stratification by province and date of birth (factors used in case-control matching), this immunization model did not include indicators $\mathbf{I}_i$ for each stratum, but instead used random terms $b_p$ for each province $p$ and natural cubic splines $\mathbf{s}(B_{ij})$ for date of birth with 5 knots at the 5th, 27.5th, 50th, 72.5th, and 95th percentiles.[4] To allow for distinct baseline immunization probabilities over time, we included indicators $\mathbf{I}(k)$ of time intervals 0, 1, 2, 3–6, 7–13, 14–29, and ≥30 days of life within the first RSV season and a single interval for the second RSV season. The model also included main terms for each category of baseline factors $\mathbf{Z}_{ij}$ and interaction terms between prevalent non-RSV hospitalization at previous day $V_{ijk\text{-}1}$ and indicators $\mathbf{I}(k)$ of aggregated time intervals 0–2 and ≥3 days of life, thus allowing for a time-dependent effect of previous non-RSV hospitalization on nirsevimab immunization. The factors in $\pi_{ijt}^{0}$ were estimated using the same sampling-weighted pooled logistic model, but with terms $\mathbf{I}(k)V_{ijk\text{-}1}$ associated with prior non-RSV hospitalization removed from the model.

Stabilized censoring weights for the ITT analysis of the two-season effectiveness of catch-up immunization were estimated in a similar way, except that children were followed up from the start of the 2023-24 immunization campaign in each Spanish region and the target immunization period lasted the first 30 days of campaign.[1] Due to the longer immunization period, the sampling-weighted pooled logistic model for the probability of remaining unimmunized after 2023-24 campaign onset included indicators $\mathbf{I}(k)$ of time intervals 0–6, 7–14, 15–29, 30–59, and ≥60 days within the first season and a single interval for the second

season as main terms, as well as aggregated intervals 0–29 and ≥30 days for interaction terms with previous non-RSV hospitalization $V_{ijk\text{-}1}$.

***Estimation of censoring weights for per-protocol analysis***

In the at-birth immunization study, the originally selected cases and their matched controls contributed to the per-protocol (PP) analysis with their uncensored clones assigned to immunization who received nirsevimab by day 14 of life without subsequent immunization until the matching date, as well as their uncensored clones assigned to no immunization who did not receive nirsevimab at any time from birth to matching date.[1] Thus, for the PP analysis, uncensored clone $c = 1$ allocated to immunization and $c = 0$ to no immunization of child $j$ in population stratum $i$ selected in a matched set for a case hospitalized at day $t$ of life was assigned the stabilized censoring weight

$$W^C_{ijct} = \begin{cases} 1, & c = 1,\ 0 \leq t \leq 13, \\ (1 - \pi^0_{ij13})/(1 - \pi^1_{ij13}), & c = 1,\ t \geq 14, \\ \pi^0_{ijt}/\pi^1_{ijt}, & c = 0,\ t \geq 0. \end{cases}$$

Note that, in the immunization group, both the numerator and denominator of $W^C_{ijct}$ should actually include an additional factor for the conditional probability of clones not being immunized again after the first 14 days of life. We omitted this factor because, among 2,838 children (522 cases and 2,316 controls) already immunized by day 14 of life, none received subsequent doses of nirsevimab during the first RSV season and only 8 children (1 case and 7 controls) received them during the second season, which precluded the conditional probability of censoring due to multiple immunization from being consistently estimated. Nevertheless, these few censorings, albeit potentially informative, were expected to introduce negligible bias in the PP analysis.

Similar stabilized censoring weights were estimated for the PP analysis of the two-season effectiveness of catch-up immunization within the first 30 days of the 2023-24

nirsevimab immunization campaign.[1] None of the 1,539 children (275 cases and 1,264 controls) immunized by day 30 of campaign received subsequent doses of nirsevimab during the 2023-24 and 2024-25 RSV seasons, so all their clones assigned to immunization remained uncensored thereafter.

**Supplementary results**

***Factors related to nirsevimab immunization***

The odds ratios for remaining unimmunized with nirsevimab by category of baseline and time-varying factors are shown in **Supplementary Tables S2 and S3**, as estimated from the above sampling-weighted pooled logistic models among previously unimmunized healthy term-born controls. In the at-birth immunization study (first columns of **Supplementary Table S3**), the odds of remaining unimmunized increased by 23% (95% CI, −8% to 65%) in late pre-term (35–36 weeks of gestation) compared with full-term newborns (≥39 weeks) and by 27% (95% CI, −4% to 69%) in newborns with low (<2,500 g) compared with normal birthweight (≥3,000 g), with no differences by sex and multiple pregnancy. Newborns with previous non-RSV hospitalization delayed nirsevimab immunization compared with those without hospitalization, with odds ratios for non-immunization of 3.56 (95% CI, 2.43 to 5.22) at 0–2 days of life and 0.68 (95% CI, 0.47 to 0.97) beyond 3 days of life.

The associations were weaker with catch-up immunization among children born before the start of the 2023-24 immunization campaign (first columns of **Supplementary Table S2**). The odds of remaining unimmunized decreased by 20% (95% CI, −39% to 6%) and 13% (95% CI, −24% to −1%) in children with low and intermediate birthweight (2,500–3,000 g) compared with normal birthweight, respectively, and increased by 26% (95% CI, −22% to 102%) in children born from multiple pregnancy, with no differences by sex and gestational age. Although immunization during the first 30 days of the 2023-24 campaign did not differ

by previous non-RSV hospitalization, the odds of remaining unimmunized later on was 25% higher (95% CI, −36% to 145%) in previously hospitalized children.

***Sampling-by-censoring weights***

For the two-season effectiveness, the mean (range) final weights (product of sampling weights $W_{ij}^{S}$ and censoring weights $W_{ijct}^{C}$) among cases and controls were, respectively, 1.00 (0.60–2.50) and 1.00 (0.54–2.50) for ITT analysis and 1.00 (0.66–2.50) and 1.00 (0.54–3.60) for PP analysis in the at-birth immunization study, and 0.99 (0.80–2.02) and 0.99 (0.79–2.02) for ITT analysis and 0.98 (0.65–2.03) and 0.97 (0.63–2.05) for PP analysis in the catch-up immunization study. These final weights provided an effective control for informative censoring due to prior non-RSV hospitalization as, given stratifying and baseline factors, these non-RSV hospitalizations were unrelated with subsequent nirsevimab immunization in the fully-weighted analyses of the at-birth and catch-up immunization studies (last columns of **Supplementary Tables S2 and S3**).

## Supplementary references


1. Núñez O, Olmedo C, Moreno-Pérez D, Lorusso N, Fernández Martínez S, Pastor Villalba PE, et al. Effectiveness of catch-up and at-birth nirsevimab immunisation against RSV hospital admission in the first year of life: a population-based case-control study, Spain, 2023/24 season. Euro Surveill. 2025;30(5):2400596. https://doi.org/10.2807/1560-7917.ES.2025.30.5.2400596. PMID: 39916606.
2. Robins JM, Hernán MA, Brumback B. Marginal structural models and causal inference in epidemiology. Epidemiology. 2000;11(5):550-60. https://doi.org/10.1097/00001648-200009000-00011. PMID: 10955408.
3. Brumback BA, Bouldin ED, Zheng HW, Cannell MB, Andresen EM. Testing and estimating model-adjusted effect-measure modification using marginal structural models and complex survey data. Am J Epidemiol. 2010;172(9):1085-91. https://doi.org/10.1093/aje/kwq244. PMID: 20801863.
4. Hernán MA, Brumback B, Robins JM. Marginal structural models to estimate the causal effect of zidovudine on the survival of HIV-positive men. Epidemiology 2000;11(5):561-70. https://doi.org/10.1097/00001648-200009000-00012. PMID: 10955409.

**Supplementary Table S1.** Selected cases hospitalized for RSV infection and sampling weights by RSV season and autonomous region in the catch-up and at-birth nirsevimab immunization studies, Spain, October 2023–March 2025.*

| | | Catch-up immunization study | | | | At-birth immunization study | | | |
|---|---|---|---|---|---|---|---|---|---|
| | | 2023-24 RSV season | | 2024-25 RSV season | | 2023-24 RSV season | | 2024-25 RSV season | |
| **Autonomous region†** | **Start of 2023-24 immunization campaign** | **Selected‡/ eligible cases** | **Sampling weight§** | **Selected‡/ eligible cases** | **Sampling weight§** | **Selected‡/ eligible cases** | **Sampling weight§** | **Selected‡/ eligible cases** | **Sampling weight§** |
| Overall | | 276/276 | | 212/320 | | 354/354 | | 348/488 | |
| Aragon | October 2 | 7/7 | 0.8188 | 11/12 | 0.8932 | 12/12 | 0.8337 | 15/23 | 1.2784 |
| Asturias | October 2 | 9/9 | 0.8188 | 11/11 | 0.8188 | 4/4 | 0.8337 | 27/27 | 0.8337 |
| Basque Country | November 13 | | | | | 9/9 | 0.8337 | 28/34 | 1.0124 |
| Canary Islands | October 5 | 34/34 | 0.8188 | 31/34 | 0.8980 | 21/21 | 0.8337 | 35/38 | 0.9052 |
| Cantabria | October 1 | 0/0 | 0.8188 | 5/5 | 0.8188 | 4/4 | 0.8337 | 1/2 | 1.6675 |
| Castile and Leon | October 1 | 9/9 | 0.8188 | 24/52 | 1.7740 | 19/19 | 0.8337 | 35/58 | 1.3816 |
| Castilla-La Mancha | October 2 | 32/32 | 0.8188 | 27/32 | 0.9704 | 37/37 | 0.8337 | 31/36 | 0.9682 |
| Catalonia | October 2 | 40/40 | 0.8188 | 33/38 | 0.9429 | 48/48 | 0.8337 | 61/71 | 0.9704 |
| Ceuta | October 6 | 2/2 | 0.8188 | 0/0 | 0.8188 | 2/2 | 0.8337 | 2/2 | 0.8337 |
| Extremadura | October 30 | | | | | 5/5 | 0.8337 | 6/7 | 0.9727 |
| Galicia | September 25 | 33/33 | 0.8188 | 25/40 | 1.3101 | 29/29 | 0.8337 | 33/58 | 1.4653 |
| La Rioja | October 1 | 4/4 | 0.8188 | 5/5 | 0.8188 | 6/6 | 0.8337 | 3/3 | 0.8337 |
| Melilla | October 16/23 | | | | | 5/5 | 0.8337 | 1/3 | 2.5012 |
| Murcia | September 25 | 21/21 | 0.8188 | 5/5 | 0.8188 | 31/31 | 0.8337 | 16/26 | 1.3548 |
| Navarre | October 1 | | | | | 14/14 | 0.8337 | 10/10 | 0.8337 |
| Valencia | October 1 | 85/85 | 0.8188 | 35/86 | 2.0119 | 108/108 | 0.8337 | 44/90 | 1.7054 |

RSV, respiratory syncytial virus.

* Data include all eligible cases during the entire 2023-24 and 2024-25 RSV seasons among both healthy term-born and high-risk children.

† Eleven regions participated with the whole public hospital network in the entire region, 2 regions (Canary Islands and Castile and Leon) participated with the public hospital network in 3 highly populated islands/provinces, and 3 regions (Aragon, Catalonia, and Extremadura) included only some public hospitals with better data accessibility. Four regions were excluded from the catch-up immunization study because their 2023-24 nirsevimab immunization campaigns began in late October or November (Basque Country, Extremadura, and Melilla) or were only implemented for children at high risk (Navarre).

‡ All eligible cases in the 2023-24 RSV season were included. In the 2024-25 RSV season, a region-stratified sample of cases up to January 31, 2025 was selected, including all cases up to that date in the 10 regions with less than 50 cases and a random sample of at least 50 cases in the remaining 6 regions.

§ Sampling weights were calculated as the inverse of the sampling fraction of cases within each study, RSV season, and region, further rescaled to the overall sampling fraction in each study.

**Supplementary Table S2.** Odds ratios for remaining unimmunized with nirsevimab by baseline and time-varying factors among previously unimmunized healthy term-born controls in the catch-up immunization study, Spain, October 2023–March 2025.

| Characteristic | Sampling-weighted odds ratio* (95% CI) | *P* value | Fully-weighted odds ratio† (95% CI) | *P* value |
|---|---|---|---|---|
| Sex | | | | |
| Female | 1.00 (reference) | | 1.00 (reference) | |
| Male | 0.93 (0.84 to 1.04) | 0.21 | 0.94 (0.84 to 1.04) | 0.23 |
| Gestational age (weeks) | | | | |
| 35–36 | 1.03 (0.74 to 1.43) | 0.88 | 1.04 (0.74 to 1.45) | 0.83 |
| 37–38 | 1.03 (0.90 to 1.17) | 0.71 | 1.03 (0.90 to 1.17) | 0.72 |
| ≥39 | 1.00 (reference) | | 1.00 (reference) | |
| Birthweight (g) | | | | |
| <2,500 | 0.80 (0.61 to 1.06) | 0.11 | 0.80 (0.60 to 1.05) | 0.11 |
| 2,500–3,000 | 0.87 (0.76 to 0.99) | 0.04 | 0.87 (0.76 to 0.99) | 0.04 |
| ≥3,000 | 1.00 (reference) | | 1.00 (reference) | |
| Multiple pregnancy | | | | |
| No | 1.00 (reference) | | 1.00 (reference) | |
| Yes | 1.26 (0.78 to 2.02) | 0.35 | 1.25 (0.78 to 2.00) | 0.36 |
| Previous non-RSV hospitalization‡ | | | | |
| 0–29 days from 2023-24 campaign onset | | | | |
| No | 1.00 (reference) | | 1.00 (reference) | |
| Yes | 1.01 (0.79 to 1.30) | 0.92 | 1.00 (0.78 to 1.29) | 0.99 |
| ≥30 days from 2023-24 campaign onset | | | | |
| No | 1.00 (reference) | | 1.00 (reference) | |
| Yes | 1.25 (0.64 to 2.45) | 0.51 | 1.01 (0.54 to 1.88) | 0.98 |

CI, confidence interval; RSV, respiratory syncytial virus.

* Sampling-weighted odds ratios for remaining unimmunized with nirsevimab (95% CIs) were obtained from a pooled logistic model among previously unimmunized healthy term-born controls, with sampling weights inversely proportional to the selection probability of their matched cases, treating each child-day as an observation, allowing for a time-dependent intercept (0–6, 7–14, 15–29, 30–59, and ≥60 days within the first RSV season and the entire second RSV season), and adjusting for matching factors (random terms for province and natural cubic splines for birthdate with knots at the 5th, 27.5th, 50th, 72.5th, and 95th percentiles) and all categorical factors listed in the table. Conservative 95% CIs and *P* values were calculated using robust standard errors.

† Fully-weighted odds ratios (95% CIs) were obtained from the same pooled logistic model with weights equal to the product of sampling weights and inverse-probability-of-immunization weights. Immunization weights were calculated as the probability of having the observed immunization history up to the current day given matching factors and baseline factors (sex, gestational age, birthweight, and multiple pregnancy), divided by the same probability further conditional on prevalent non-RSV hospitalization at previous day.

‡ Odds ratios for previous non-RSV hospitalization varied over time and were modeled by including interaction terms between this factor and time interval indicators (0–29 and ≥30 days from 2023-24 campaign onset).

**Supplementary Table S3.** Odds ratios for remaining unimmunized with nirsevimab by baseline and time-varying factors among previously unimmunized healthy term-born controls in the at-birth immunization study, Spain, October 2023–March 2025.

| Characteristic | Sampling-weighted odds ratio* (95% CI) | *P* value | Fully-weighted odds ratio† (95% CI) | *P* value |
|---|---|---|---|---|
| Sex | | | | |
| Female | 1.00 (reference) | | 1.00 (reference) | |
| Male | 0.98 (0.88 to 1.09) | 0.71 | 0.96 (0.87 to 1.07) | 0.48 |
| Gestational age (weeks) | | | | |
| 35–36 | 1.23 (0.92 to 1.65) | 0.16 | 1.31 (0.97 to 1.76) | 0.08 |
| 37–38 | 1.08 (0.94 to 1.23) | 0.27 | 1.08 (0.95 to 1.23) | 0.25 |
| ≥39 | 1.00 (reference) | | 1.00 (reference) | |
| Birthweight (g) | | | | |
| <2,500 | 1.27 (0.96 to 1.69) | 0.09 | 1.29 (0.98 to 1.69) | 0.07 |
| 2,500–3,000 | 0.96 (0.83 to 1.10) | 0.55 | 0.95 (0.82 to 1.09) | 0.43 |
| ≥3,000 | 1.00 (reference) | | 1.00 (reference) | |
| Multiple pregnancy | | | | |
| No | 1.00 (reference) | | 1.00 (reference) | |
| Yes | 1.06 (0.73 to 1.53) | 0.78 | 1.00 (0.70 to 1.44) | 0.99 |
| Previous non-RSV hospitalization‡ | | | | |
| 0–2 days of life | | | | |
| No | 1.00 (reference) | | 1.00 (reference) | |
| Yes | 3.56 (2.43 to 5.22) | <0.001 | 0.97 (0.69 to 1.37) | 0.88 |
| ≥3 days of life | | | | |
| No | 1.00 (reference) | | 1.00 (reference) | |
| Yes | 0.68 (0.47 to 0.97) | 0.03 | 1.08 (0.58 to 2.01) | 0.80 |

CI, confidence interval; RSV, respiratory syncytial virus.

* Sampling-weighted odds ratios for remaining unimmunized with nirsevimab (95% CIs) were obtained from a pooled logistic model among previously unimmunized healthy term-born controls, with sampling weights inversely proportional to the selection probability of their matched cases, treating each child-day as an observation, allowing for a time-dependent intercept (0, 1, 2, 3–6, 7–13, 14–29, and ≥30 days of life within the first RSV season and the entire second RSV season), and adjusting for matching factors (random terms for province and natural cubic splines for birthdate with knots at the 5th, 27.5th, 50th, 72.5th, and 95th percentiles) and all categorical factors listed in the table. Conservative 95% CIs and *P* values were calculated using robust standard errors.

† Fully-weighted odds ratios (95% CIs) were obtained from the same pooled logistic model with weights equal to the product of sampling weights and inverse-probability-of-immunization weights. Immunization weights were calculated as the probability of having the observed immunization history up to the current day given matching factors and baseline factors (sex, gestational age, birthweight, and multiple pregnancy), divided by the same probability further conditional on prevalent non-RSV hospitalization at previous day.

‡ Odds ratios for previous non-RSV hospitalization varied over time and were modeled by including interaction terms between this factor and time interval indicators (0–2 and ≥3 days of life).

**Supplementary Table S4.** Selected cases hospitalized for RSV infection and density-matched population controls by RSV season and autonomous region among healthy term-born children in the catch-up nirsevimab immunization study, Spain, October 2023–March 2025.*

| | 2023-24 RSV season | | 2024-25 RSV season | | Both RSV seasons | |
|---|---|---|---|---|---|---|
| **Autonomous region** | **Cases** | **Controls** | **Cases** | **Controls** | **Cases** | **Controls** |
| Aragon | 6 (2.6) | 23 (2.5) | 11 (4.3) | 43 (4.5) | 17 (3.5) | 66 (3.6) |
| Asturias | 6 (2.6) | 24 (2.7) | 10 (3.6) | 39 (3.7) | 16 (3.1) | 63 (3.2) |
| Canary Islands | 27 (11.5) | 103 (11.4) | 27 (10.6) | 102 (10.6) | 54 (11.0) | 205 (11.0) |
| Cantabria | 0 (0.0) | 0 (0.0) | 5 (1.8) | 20 (1.9) | 5 (1.0) | 20 (1.0) |
| Castile and Leon | 9 (3.8) | 35 (3.9) | 20 (15.5) | 77 (15.8) | 29 (10.2) | 112 (10.3) |
| Castilla-La Mancha | 30 (12.8) | 113 (12.5) | 25 (10.6) | 98 (11.0) | 55 (11.6) | 211 (11.7) |
| Catalonia | 38 (16.2) | 144 (15.9) | 29 (12.0) | 113 (12.4) | 67 (13.9) | 257 (14.0) |
| Ceuta | 1 (0.4) | 4 (0.4) | 0 (0.0) | 0 (0.0) | 1 (0.2) | 4 (0.2) |
| Galicia | 28 (11.9) | 110 (12.2) | 24 (13.8) | 83 (12.6) | 52 (12.9) | 193 (12.4) |
| La Rioja | 4 (1.7) | 16 (1.8) | 5 (1.8) | 20 (1.9) | 9 (1.8) | 36 (1.8) |
| Murcia | 18 (7.7) | 69 (7.6) | 4 (1.4) | 14 (1.3) | 22 (4.3) | 83 (4.2) |
| Valencia | 68 (28.9) | 264 (29.2) | 28 (24.6) | 104 (24.3) | 96 (26.6) | 368 (26.5) |

RSV, respiratory syncytial virus.
* Data are unweighted counts (sampling-weighted percentages).

**Supplementary Table S5.** Selected cases hospitalized for RSV infection and density-matched population controls by RSV season and autonomous region among healthy term-born children in the at-birth nirsevimab immunization study, Spain, October 2023–March 2025.*

| | **2023-24 RSV season** | | **2024-25 RSV season** | | **Both RSV seasons** | |
|---|---|---|---|---|---|---|
| **Autonomous region** | **Cases** | **Controls** | **Cases** | **Controls** | **Cases** | **Controls** |
| Aragon | 12 (3.6) | 46 (3.6) | 15 (5.0) | 59 (5.1) | 27 (4.4) | 105 (4.4) |
| Asturias | 2 (0.6) | 6 (0.5) | 25 (5.4) | 94 (5.3) | 27 (3.4) | 100 (3.3) |
| Basque Country | 9 (2.7) | 36 (2.8) | 27 (7.1) | 106 (7.2) | 36 (5.3) | 142 (5.4) |
| Canary Islands | 20 (6.0) | 74 (5.7) | 34 (8.0) | 135 (8.2) | 54 (7.2) | 209 (7.2) |
| Cantabria | 4 (1.2) | 16 (1.2) | 1 (0.4) | 4 (0.5) | 5 (0.8) | 20 (0.8) |
| Castile and Leon | 19 (5.7) | 76 (5.9) | 35 (12.6) | 139 (13.0) | 54 (9.7) | 215 (10.0) |
| Castilla-La Mancha | 34 (10.2) | 133 (10.3) | 28 (7.1) | 110 (7.2) | 62 (8.4) | 243 (8.5) |
| Catalonia | 46 (13.8) | 173 (13.4) | 57 (14.4) | 218 (14.3) | 103 (14.2) | 391 (13.9) |
| Ceuta | 1 (0.3) | 4 (0.3) | 1 (0.2) | 4 (0.2) | 2 (0.3) | 8 (0.3) |
| Extremadura | 5 (1.5) | 19 (1.5) | 5 (1.3) | 18 (1.2) | 10 (1.4) | 37 (1.3) |
| Galicia | 27 (8.1) | 105 (8.1) | 32 (12.2) | 119 (11.8) | 59 (10.5) | 224 (10.2) |
| La Rioja | 6 (1.8) | 24 (1.9) | 3 (0.7) | 12 (0.7) | 9 (1.1) | 36 (1.2) |
| Melilla | 5 (1.5) | 20 (1.5) | 1 (0.7) | 4 (0.7) | 6 (1.0) | 24 (1.0) |
| Murcia | 30 (9.0) | 116 (9.0) | 15 (5.3) | 57 (5.2) | 45 (6.9) | 173 (6.8) |
| Navarre | 14 (4.2) | 56 (4.3) | 10 (2.2) | 40 (2.3) | 24 (3.0) | 96 (3.1) |
| Valencia | 100 (29.9) | 388 (30.0) | 39 (17.4) | 150 (17.3) | 139 (22.6) | 538 (22.6) |

RSV, respiratory syncytial virus.
* Data are unweighted counts (sampling-weighted percentages).
